# Semantic Adaptation to the Interpretation of Gradable Adjectives via Active Linguistic Interaction

**DOI:** 10.1111/cogs.13248

**Published:** 2023-02-05

**Authors:** Sandro Pezzelle, Raquel Fernández

**Affiliations:** ^1^ Institute for Logic, Language and Computation University of Amsterdam

**Keywords:** Gradable adjectives, Semantic adaptation, Vagueness, Questions, Linguistic interaction, Visual grounding

## Abstract

When communicating, people adapt their linguistic representations to those of their interlocutors. Previous studies have shown that this also occurs at the semantic level for vague and context‐dependent terms such as quantifiers and uncertainty expressions. However, work to date has mostly focused on passive exposure to a given speaker's interpretation, without considering the possible role of active linguistic interaction. In this study, we focus on gradable adjectives *big* and *small* and develop a novel experimental paradigm that allows participants to ask clarification questions to figure out their interlocutor's interpretation. We find that, when in doubt, speakers do resort to this strategy, despite its inherent cognitive cost, and that doing so results in higher semantic alignment measured in terms of communicative success. While not all question–answer pairs are equally informative, we show that speakers become better questioners as the interaction progresses. Yet, the higher semantic alignment observed when speakers are able to ask questions does not increase over time. This suggests that conversational interaction's key advantage may be to boost coordination without committing to long‐term semantic updates. Our findings shed new light on the mechanisms used by speakers to achieve semantic alignment and on how language is shaped by communication.

## 1 Introduction

Human communication is a fundamentally cooperative process (Grice, 1957, 1975), which operates within the context of mutually assumed common conceptual ground and cooperative communicative motives (Tomasello, 2010). Since speakers' primary goal is to achieve communicative success, they can resort to strategies that allow them to align their language representations to those by their interlocutors. The process leading to alignment is typically referred to as *language adaptation*. Previous work has shown that speakers can adapt to an individual's representations at many levels: by increasing the use of syntactic structures of the types frequently used by their interlocutor (Branigan, Pickering, & Cleland, 2000; Jaeger & Snider, 2013; Kamide, 2012); by adjusting and recategorizing their phonetic and phonological representations (Kraljic & Samuel, 2005; Kleinschmidt & Jaeger, 2015; Norris, McQueen, & Cutler, 2003); by mimicking speaker‐specific patterns of prosodic cues to pragmatic meanings (Kurumada, Brown, & Tanenhaus, 2012; Roettger & Franke, 2019). As for semantics, classic work on conceptual pacts found that, when referring to an entity in a specific context, interlocutors converge onto temporary, shared referential expressions (Brennan & Clark, 1996; Metzing & Brennan, 2003).

More recently, a few studies investigated whether semantic adaptation is in place for words other than nouns, such as quantifiers and expressions of uncertainty (S. Heim, Peiseler, & Bekemeier, 2020; Schuster & Degen, 2019, 2020; Yildirim, Degen, Tanenhaus, & Jaeger, 2016). Since different speakers may have different interpretations (i.e., semantic representations) of what *many* or *probably* denote in a given context, adapting to an interlocutor's semantic representation of these words is needed to achieve alignment and therefore communicative success. By focusing on the quantifiers *some* and *many*, Yildirim et al. (2016) showed that listeners can indeed adapt to a specific talker in terms of both the frequency of use and the semantic interpretation of these expressions. After being exposed to an individual's interpretation of *some* and *many* in a visual context, listeners are shown to mimic that interpretation—for example, that *many* denotes at least n% dots in the image—when asked to use the quantifiers as the speaker would do. This reveals they can adapt their representations to align them to those by another speaker. Similar evidence was reported by S. Heim et al. (2020) for the vague quantifiers *few* and *many*, while Schuster and Degen (2019) reported a comparable adaptation mechanism for the expressions of uncertainty *might* and *probably*. Recently, Schuster and Degen (2020) further showed that this adaptation is best captured by speakers who update beliefs both about the interlocutor's lexicon and their utterance preferences.

Taken together, these studies show that semantic adaptation takes place while being exposed to an individual's interpretation of quantifiers and expressions of uncertainty. Importantly, in these studies, a listener is only given the possibility to learn by passively observing the linguistic behavior of a speaker, while there is no active dialogical interaction between the listener and the speaker. However, being passively exposed to someone else's interpretation might not be the only—nor perhaps the most effective—mechanism by which people can adapt in real‐life contexts. Consider the following example involving gradable adjectives *big* and *small*. Suppose you are on your first day of work at a restaurant, and the chef asks you to butter a *small* pan. If all the pans in the kitchen look rather *big* to you, you may take this statement as proof that there are indeed some pans that are considered as *small* by the chef and decide to butter one—likely the smallest. Alternatively, to have more direct evidence and learn once and for all when a pan counts as *small* in that kitchen, you may decide to ask the chef if the pan that you have been using earlier that day can be a good one; if there is more than one such pan in the kitchen; and so on. As the example highlights, actively seeking information by asking clarification questions could be beneficial to improve alignment and thus the communicative success of two interlocutors. This would be in line with the more general patterns involving *grounding* and *semantic coordination* in dialogue (Larsson, 2018), where so‐called “repair events,” including clarification requests, are very frequent particularly in task‐oriented interactions (Colman & Healey, 2011, e.g., report an average of one such event every 2.5 dialogue turns). The questions of whether speakers do ask questions to achieve semantic adaptation, and the extent to which this strategy brings any improvement, however, have not been explored to date. By focusing on gradable adjectives *big* and *small*, we are the first to investigate the utility of asking questions for semantic adaptation.

Asking questions is a core cognitive and linguistic tool to gain information and learn about the world. Indeed, a wealth of studies have highlighted the role of asking questions in language acquisition (Nelson, 1973), children's information search (Ruggeri, Lombrozo, Griffiths, & Xu, 2016), and efficient communication (Hawkins, Stuhlmüller, Degen, & Goodman, 2015), very often with a focus on the *utility* of the questions with respect to a given goal (Coenen, Nelson, & Gureckis, 2019). Rothe, Lake, and Gureckis (2018), for example, explored whether people ask *good* questions when discovering the configuration of the ships in a battleship gameboard. By measuring question informativeness through a Bayesian model, they showed that people have limited ability to ask questions that are maximally informative. However, their experimental setup did not allow for the possibility of testing the impact of asking a question on task performance: indeed, participants did not complete the game based on the answer. This aspect was instead crucial in the setting by Hawkins et al. (2015), where a questioner had to communicate with an answerer to be successful in guessing objects. In doing so, learners were shown to ask questions that were sensitive to pragmatic aspects, that is, they took into account the context or state of the world shared with the answerer. This complements previous evidence that, while having an interaction aimed at achieving a goal, participants exploit alignment to resolve differences in their underlying semantic models (e.g., the different ways in which they describe a maze; see Mills & Healey, 2008).

These findings indicate that actively seeking information is beneficial to tasks where some underlying representations need to be discovered. Consistently, we hypothesize that asking questions during linguistic interaction will have a positive impact on semantic adaptation since it facilitates understanding the underlying semantic representation governing a speaker's interpretation. We, therefore, conjecture that, when given the possibility, participants do ask clarification questions, and that this strategy leads to improved alignment—and thus higher communicative success—compared to the setting where this possibility is not given. At the same time, we expect that asking well is difficult, and not all questions will be equally informative.

To test our hypotheses, we build on previous work on semantic adaptation (S. Heim et al., 2020; Schuster & Degen, 2019, Yildirim et al., 2016) and focus on a still unexplored (in the domain of adaptation) class of words: gradable adjectives. Gradable adjectives such as *big*, *tall*, *young*, or *cold* are interesting for various reasons. First, their meaning is relative, that is, determined by the context; for example, Frodo Baggins might count as *tall* in some circumstances (a *tall* hobbit), as *short* in others (a *short* character of the *Lord of the Rings*).^1^ According to most semantic analyses (Cresswell, 1977; I. Heim, 2000; Kennedy, 1999, 2007; Kennedy & McNally, 2005, among others), gradable adjectives denote functions that map their arguments onto abstract representations of measurement, or *degrees*. Since a set of degrees that is ordered with respect to some dimension constitutes a *scale*, using adjectives such as *tall* or *big* implies mapping a target entity onto its corresponding scale, for example, the scale of height or size, respectively, which in turn makes it possible to use comparative and superlative degree morphology to express ordering relations between entities in the scale, for example, *taller* or *biggest*. The semantics of gradable adjectives in their basic, positive form (e.g., *tall*) would therefore depend on the relation between the degree to which an object has some gradable concept (e.g., height) and a context‐dependent standard of comparison (Kennedy, 2007). Some classical work (Bartsch & Vennemann, 1972) proposed that the actual value of the context‐dependent standard of comparison can be operationalized in terms of a *threshold* obtained by applying some statistical function over the relevant context. For example, Frodo would count as a *tall* hobbit if his height exceeds the average height of the hobbits. This is in line with evidence showing that children are sensitive to the statistics of sets when using gradable adjectives like *tall* (Barner & Snedeker, 2008). More recently, a systematic investigation of various possible statistical functions was carried out by Schmidt, Goodman, Barner, and Tenenbaum (2009) and Solt and Gotzner (2012), while Qing and Franke (2014a, 2014b) and Lassiter and Goodman (2013, 2017) proposed more complex probabilistic models which also account for pragmatic reasoning aspects driving the use of these expressions.

Second, besides being context‐dependent, gradable adjectives are vague, that is, they admit borderline cases (Lassiter, 2011; Lassiter & Goodman, 2013; Van Deemter, 2012). Context‐dependence and vagueness are not necessarily related concepts: the context may have been fixed, and yet the threshold for accepting a statement as true can be fuzzy (Solt, 2011). For example, even if a speaker knows that Frodo is taller than the average hobbit, they may still be hesitant in considering him as *tall*. At the same time, borderline cases appear to be more likely around the context‐dependent threshold, at least according to probabilistic approaches (Lassiter & Goodman, 2013). Consistently, vagueness could be seen as a source of noise added to the decision process, with noise being higher around a speaker's threshold.

Third, gradable adjectives are subject to speaker variability, that is, various speakers can differ in the subjective way they use these expressions even when the context parameters (i.e., the comparison class, the standard of comparison, etc.) are fixed (Égré, 2017; Verheyen, Dewil, & Égré, 2018; Verheyen & Égré, 2018). As reported by Verheyen et al. (2018), for adjectives that can relate to measurements of human bodies such as *tall* and *heavy*, subjectivity may be due in part to people using themselves as a “yardstick” and therefore grounding judgments on their personal measurements. In this light, the interpretation of gradable adjectives would depend not only on a comparison class and a standard of comparison but also on a given *perspective*. More in general, Solt (2018) proposed that the observed variability in the use of gradable adjectives would result from the speaker‐ and context‐dependent weighting of the multiple dimensions that underlie many gradable adjectives. As a consequence, various speakers may have a different interpretation of what count as *tall*, *small*, or *heavy* in a given context, similarly to what has been reported for quantifiers (S. Heim et al., 2020; Yildirim et al., 2016) and expressions of uncertainty (Schuster & Degen, 2019). All these aspects—context‐dependence, vagueness, and speaker variability—make gradable adjectives an interesting benchmark for testing semantic adaptation.

We experiment with one specific pair of terms, *big* and *small*, and conduct two behavioral experiments with 60 participants.^2^ We use data from the recent Modeling Adjectives Leveraging Visual Contexts (MALeViC) dataset (Pezzelle & Fernández, 2019), which contains synthetic images depicting colored geometric objects paired with statements about the size category of one object. Crucially, in this dataset, objects are deemed *big* or *small* based on their own area and that of the surrounding objects; that is, an object that counts as *big* in one context can be *small* in another context. This type of data is similar to that used by experimental work in developmental psychology that explores children's interpretation of gradable adjectives (Barner & Snedeker, 2008).

**Fig. 1:**
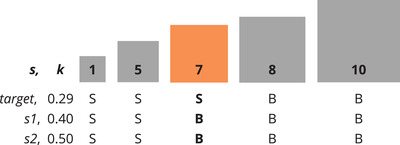
Toy illustration of a reference set with five squares of area 1, 5, 7, 8, and 10 units and corresponding *big*/*small* (B/S) interpretation by three speakers *(s)*. All three toy speakers use a threshold function T=Max−k(Max−Min), where Max and Min are the largest and smallest areas in the reference set and *k* defines the k% of the range of sizes counting as *big* (Schmidt et al., 2009). The *target* speaker uses k=0.29, which was shown to be the overall best‐predictive *k* by Schmidt et al. (2009). According to the *target* speaker, the orange square is a *borderline* case of *small*, while both *s1* (k=0.4) and *s2* (k=0.5) consider it as *big*. No disagreement is observed for the gray (*clear‐cut*) squares.

In Experiment 1 (20 participants), we preliminarily test whether, and to what extent, speakers have different interpretations of what counts as *big* and *small* in a given visual context. We consider one specific threshold function (the best‐predictive of human behavior reported by Schmidt et al., 2009) and quantify participants' degree of alignment, measured in terms of matching judgments, with the interpretation resulting from this function. We refer to this best‐predictive threshold function as the *target* one and to the ideal speaker systematically relying on it as our *target* speaker (see caption of Fig. 1). We test two conditions: one including cases that are *clear‐cut* according to *target*, that is, far from the statistically‐defined threshold; one including cases that are *borderline*, that is, close to the threshold.

As shown in Fig. 1, participants relying on slightly different values of *k* compared to *target* ^3^ may still agree with it when judging the size of cases that are *clear‐cut* for *target* (gray). In contrast, a slightly different *k* may lead to a different judgment for cases deemed *borderline* (orange). As a consequence, we hypothesize that *borderline* cases should exhibit lower alignment between participants and *target* compared to *clear‐cut* ones. Our results confirm this hypothesis: While alignment is significantly higher than chance for *clear‐cut* cases, this is not the case for *borderline* cases. Moreover, the agreement between participants in the former group is significantly higher than in the latter. These findings reveal that semantic variation is at work: Different speakers involved in a conversation might, in some cases, refer to the same object using two different adjectives. In these cases, semantic adaptation may be required to achieve communicative success.

In Experiment 2 (40 participants), we focus on similar *borderline* cases and investigate whether semantic adaptation is in place for *big/small* similarly to quantifiers and expressions of uncertainty. We use a game‐like experimental setting where the participants' goal is to achieve communicative success with a target speaker, that is, to interpret *big* and *small* as the target speaker would do. As such, the experiment is interactive: after each trial, participants receive feedback (correct/wrong) that can be exploited in the following trial. We experiment with two conditions: one in which speakers are given the possibility to ask clarification questions to the target speaker before assessing the trial (Q); one in which they can only learn by passively interacting with it (C). This latter setting is loosely comparable to that by Yildirim et al. (2016), S. Heim et al. (2020), and Schuster and Degen (2019).

We hypothesize that semantic adaptation is in place for gradable adjectives in both C and Q. Moreover, we conjecture that participants in Q will actively interact with the target speaker by asking clarification questions and that this will lead to higher semantic alignment—measured in terms of higher communicative success—compared to the passive condition. Our hypotheses are confirmed. While semantic adaptation is shown to take place both in C and Q, communicative success is higher in Q at any stage of the interaction. People do ask questions when in doubt, despite their cognitive cost, and this leads to higher semantic alignment compared to the passive setting. Finally, we show that question–answer pairs are not all equally informative, and people become better questioners as the interaction progresses.

The remainder of the paper is organized as follows: in Sections 2 and 3, we describe Experiments 1 and 2, respectively. In Section 4, we present analyses of the question types asked by the participants during Experiment 2. In Section 5, we discuss our methodological choices and propose several directions for future work. Finally, in Section 6, we summarize our results and their implications.

## 2 Experiment 1: Alignment with a fixed semantic interpretation

### 2.1 Materials

**Fig. 2:**
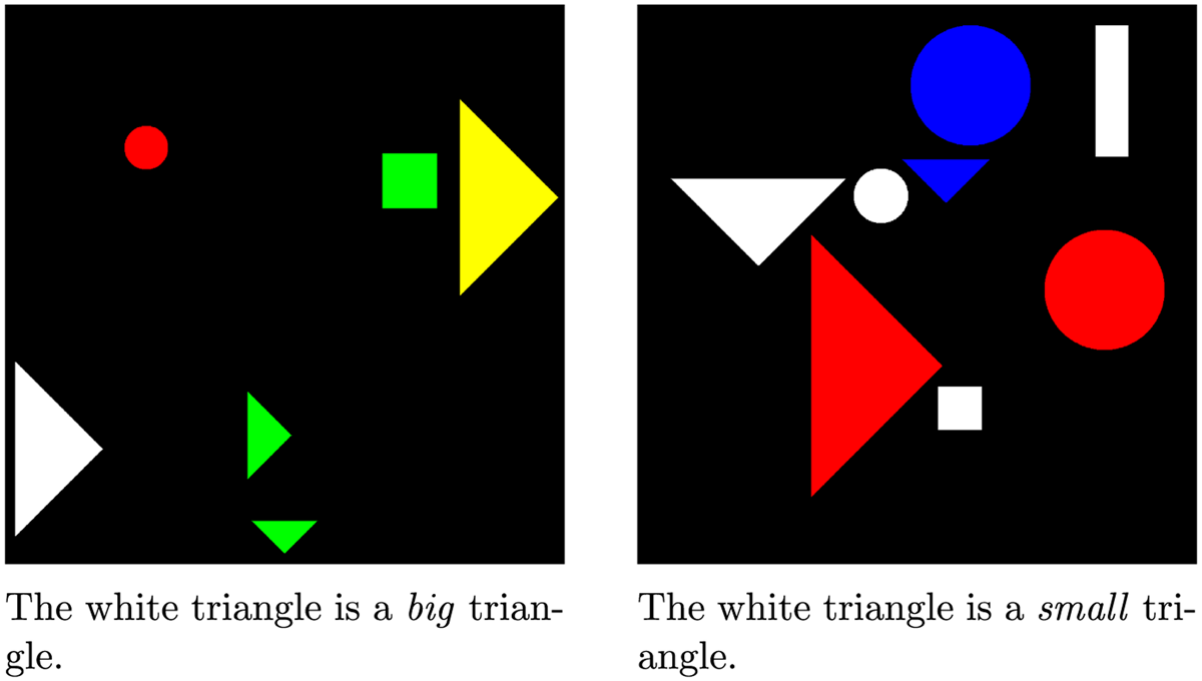
Two scenes and corresponding *true* statements from the SET+POS‐hard split of the MALeViC dataset (Pezzelle & Fernández, 2019). In both scenes, the target object is the white triangle and the reference set is the set of triangles. Note that the white triangle has the exact same area in both scenes. However, based on the different visual context (i.e., the areas of the other triangles in the reference set), it counts as *big* in the leftmost image and as *small* in the rightmost one. Best viewed in color.

We use the recent MALeViC dataset (Pezzelle & Fernández, 2019), which contains images of colored geometric shapes paired with a statement regarding the size of one object unequivocally identifiable on the basis of color, for example, *The white triangle is a big/small triangle* (see examples in Fig. 2). These statements, automatically generated during the creation of the dataset are deemed either true or false based on a threshold function *T* computed at the image level, and particularly at the level of the relevant set of objects. As a consequence, being big or small for an object uniquely depends on the visual *context*, namely its actual perceptual area in relation to that of the other relevant objects depicted in the image (e.g., the triangles). Crucially, this implies that an object with a certain area can be *big* in one scene and *small* in a different one (this is the case of the white triangle in the two scenes of Fig. 2). The function, taken from previous work investigating *tall* (Schmidt et al., 2009), considers (*i*) the area measured in terms of the number of pixels (and thus treated as a one‐dimensional property) of the largest (Max) and smallest (Min) objects in a reference set (RS) and (ii) an experimentally derived *k* which determines the top k% of the range of areas in RS that count as *big*. For each image, T(RS) is obtained as follows:

(1) T(RS)=Max−k(Max−Min),

where *k* is a value sampled from a normal distribution centered on k=0.29,^4^the best predictive value in Schmidt et al. (2009). Objects whose area exceeds the threshold are assigned the size label *big*; otherwise, they are labeled as *small* (see also Fig. 1). As acknowledged by Pezzelle and Fernández (2019), considering as *small* any object that is “not *big*” is a simplified setup, which, however, has the advantage of not having to deal with negative statements.^5^

We consider a partition of the dataset, SET+POS‐hard, where all statements are of the type *The white triangle is a big triangle*, the reference set RS is a strict subset of all objects (i.e., only includes the triangles), and the target object is neither the smallest nor the biggest in RS. We select 20 ⟨image,statement⟩ pairs (hence, trials) to be used in our experiment: 10 *clear‐cut* and 10 *borderline* cases according to the target semantic interpretation. This selection is made based on two criteria: (*i*) *k* is either equal to 0.29 or, if different, it gives rise to the same interpretation of *big/small* resulting from using k=0.29 (this is because we want to mimic the use of these adjectives by a specific speaker with a fixed underlying semantic function); (ii) the *distance from the threshold (dft)* value provided in the source dataset for each ⟨image,statement⟩ pair. Such value quantifies the normalized distance between the target object's area and *T*: the higher the *dft* value, the higher the distance from the threshold, that is, the *big/small* decision boundary. After verifying that in the entire SET+POS‐hard split the target objects' *dft* values range from 0 to 0.84, we sample 10 cases whose *dft* is <0.21 and therefore belong to the lowest quarter of the *dft* values range. We consider these 10 cases as our *borderline* cases. Similarly, we sample 10 cases whose *dft* is ≥0.21 and consider them as our *clear‐cut* cases. Based on this criterion, for example, the leftmost case in Fig. 2 would be considered as a *borderline* (dft=0.03) case of *big*, while the rightmost case would count as a *clear‐cut* (dft=0.43) case of *small*. It is worth mentioning that there are other options to turn the continuous range of *dft* values observed in the dataset into *n* discrete classes. At the same time, experimenting with two adjacent bins based on a data‐driven threshold, as we do, appears to be a reasonable starting point for any further investigation.

While selecting the 20 trials, we ensure that an equal number of *big/small* cases are present in each condition and that each of the four shape types in the MALeViC dataset (circle, rectangle, square, triangle) is equally represented. As for the five colors (blue, green, red, white, yellow), we make sure all of them are present at least once in both conditions. Descriptive statistics of the entire set of trials and the two conditions are reported in Table 1.

**Table 1:** Descriptive statistics of the images used in Experiment 1, also reported by condition. Abbreviations: (1) target object's distance from threshold; (2) number of total objects in the image; (3) number of objects in the reference set (RS); (4) number of objects in the RS with the same size as the target object (target object included); (5) number of objects in the RS with a different size compared to the target object; (6) number of total colors in the image. Note that only target *dft* (1) differs significantly from one condition to another, while the other variables do not

	All			Clear‐Cut			Borderline		
	Min.	Max.	Mean ± *SD*	Min.	Max.	Mean ± *SD*	Min.	Max.	Mean ± *SD*
1. target *dft*	0.03	0.68	0.24 ± 0.20	0.21	0.68	0.39 ± 0.18	0.03	0.17	0.08 ± 0.05
2. #objs_IMG	5	9	6.50 ± 1.40	5	8	6.10 ± 1.10	5	9	6.90 ± 1.60
3. #objs_RS	3	5	3.75 ± 0.64	3	5	3.80 ± 0.63	3	5	3.70 ± 0.67
4. #sameS_IMG	2	3	2.30 ± 0.47	2	3	2.30 ± 0.48	2	3	2.30 ± 0.48
5. #diffS_RS	1	2	1.40 ± 0.50	1	2	1.50 ± 0.53	1	2	1.30 ± 0.48
6. #colors_IMG	2	5	4.00 ± 0.79	3	5	3.90 ± 0.57	2	5	4.10 ± 0.99

### 2.2 Method

We collect data by means of a survey powered by Google Forms.^6^ In the survey, participants are shown 20 images and asked to judge, based on their standard use and interpretation of the words *big* and *small*, whether a given object depicted in the image is described better by one or the other adjective. For each trial, the question is: *How would you judge the* ⟨color,shape⟩, *as a big or a small* ⟨shape⟩? Participants are instructed to treat each trial independently—not in relation to previous/following trials. Moreover, to help them binarize their choice, they are given the hint to imagine they have two buckets where to put the big and small objects of a certain shape (e.g., big/small squares), respectively, and have to put the target object into one of them. To prevent participants from searching for any sort of underlying strategy, they are explicitly told there are neither wrong answers nor rules to discover. To perform the task, participants look at the question and the image and click on one of the two adjectives which are always presented in alphabetical order below the image. Since the aim of the experiment is to test whether, and to what extent, participants are aligned with a fixed interpretation of *big/small*, no intermediate or final feedback on their answers is provided during the survey. There is no time limit to complete the task.

We collected valid data from 20 participants. Trials were presented one below the other in a randomized order. In total, 400 datapoints (20 participants × 20 trials) were collected. All participants were proficient in English and were recruited among current or former university students and researchers. They participated on a voluntary basis. No personal data besides their contact information were collected.

### 2.3 Analysis and results

**Fig. 3:**
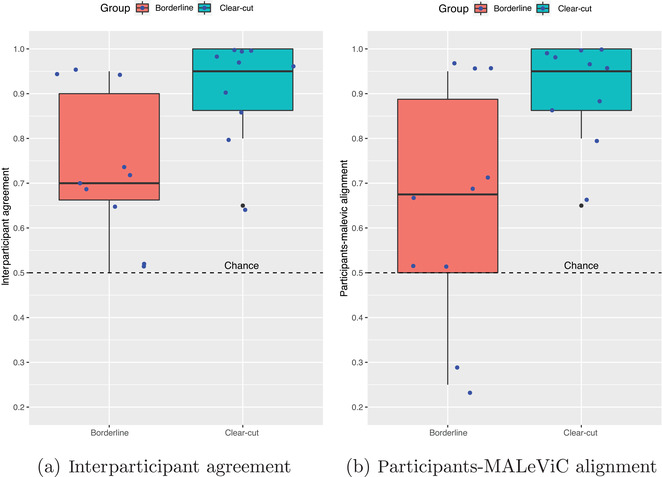
Boxplots reporting interparticipant agreement and participants‐MALeViC alignment in *borderline* and *clear‐cut* trials. Blue dots indicate the normalized frequency for each trial. Note that dots are slightly shifted from their actual value to avoid overlaps. Best viewed in color.

We preliminarily test the degree to which participants (dis)agree on the size adjective to assign to the target object in each trial. To do so, for each trial we count the number of participants who use either of the size adjectives and divide these counts by the total number of participants, that is, 20. This way, we obtain the normalized frequency, in the range [0,1], of each size label. We consider the highest value of the two and take it as a proxy for the *agreement* between speakers on a given trial. Fig. 3a reports the distribution of these agreement values in the two groups of trials. In the *clear‐cut* group, we observe an extremely high agreement between speakers, with an average of 0.91 (SD=0.11) over trials. In the *borderline* one, we observe much lower agreement: 0.74 (SD=0.17) on average. By means of a one‐sided non‐parametric Wilcoxon rank sum test,^7^ we verify that the agreement in the *clear‐cut* trials is significantly higher (Mdn=0.95) than in *borderline* ones (Mdn=0.70), W=80.5, p=.011. Consistently, we notice that, for four *clear‐cut* trials out of 10, all 20 participants agree on the same size label; in contrast, none of the *borderline* trials show such a perfect agreement. Similarly, an agreement of 0.9 or higher is observed in seven out of 10 *clear‐cut* trials, but only in three *borderline* ones. This indicates that, while people have an almost perfect agreement on *clear‐cut* cases, they largely disagree on *borderline* ones, and this difference is statistically significant.

Second, we explore whether, and to what extent, participants agree with one specific, fixed interpretation of *big/small*, namely the one used in the MALeViC dataset (i.e., the *target* interpretation in Fig. 1). To do so, for each trial we count the number of participants who use the same adjective used in MALeViC and normalize this number to range in [0,1]. We take this value as a proxy for participants' *alignment* with the target interpretation (Fig. 3b). We observe that this value matches interparticipant *agreement* in all *clear‐cut* trials; that is, for all these trials the adjective used in MALeViC is systematically chosen by the majority of participants. In contrast, this is the case for only six out of 10 trials in the *borderline* group: in two trials people converge on the opposite size, while there is a tie in the remaining two. As a result, in *borderline* trials, participants' average alignment with the MALeViC size is 0.65 (SD=0.26). We test whether this alignment is significantly greater than *chance*, that is, 0.5: by means of a one‐sided one sample *t*‐test,^8^ we verify this is not the case, t(9)=1.77, p=.110. In contrast, this hypothesis is confirmed for *clear‐cut* trials via a one‐sided Wilcoxon signed rank test, V=55, p=.006. This reveals that, when judging the size category of objects whose area is far enough from the threshold, participants are strongly aligned with the MALeViC interpretation. In contrast, people do not align with it significantly better than chance when objects are extremely close to the threshold.

### 2.4 Discussion

Taken together, these results allow us to draw two main conclusions. First, *borderline* cases have significantly lower agreement compared to *clear‐cut* ones: interlocutors can have different interpretations of what counts as *big/small* and therefore possibly use opposite adjectives to refer to the same object in these and similar c‐ases. Second, the extent to which participants align with a fixed interpretation of *big/small* in *borderline* cases is not significantly greater than chance. Since this could intuitively lead to errors and misunderstandings, speakers might resolve this disagreement by adapting to their interlocutor, that is, by inferring and subsequently using the underlying semantic function subtending the other's interpretation of *big/small*.

We hypothesize that, in real‐life communicative scenarios, this can be achieved both by being *passively* exposed to the interlocutor's interpretation—similarly to what is observed for quantifiers (S. Heim et al., 2020; Yildirim et al., 2016) and expressions of uncertainty (Schuster & Degen, 2019)—and by means of an *active* dialogical interaction, namely by asking clarification questions to the interlocutor. In particular, we conjecture that asking questions should lead to higher *alignment* compared to the passive setting, thus improving communicative success. In what follows, we test our hypotheses by means of a second experiment focused on MALeViC's *borderline* cases.

## 3 Experiment 2: Semantic adaptation via active interaction

### 3.1 Materials

We use the same partition of the MALeViC data described in Section 2.1. We select 32 ⟨image,statement⟩ pairs (hence, trials) to obtain a balanced distribution: four trials for each of the eight ⟨size,shape⟩ combinations and a balanced number of true/false statements. To focus on MALeViC's genuine *borderline* cases, these trials are selected from a subset of the data where target objects' dft<0.21 (analogously to Experiment 1). Moreover, we ensure that (*i*) the underlying semantic function which defines what counts as *big* or *small* is kept fixed across the trials (i.e., it mimics the interpretation of a specific speaker; see Section 2.1); and that (ii) variability is present among the selected scenes with respect to the number of total objects in the scene/reference set, the number of objects in the reference set with the same/different shape as the target object, and the number of unique colors in the image. Descriptive statistics are reported in Table 2. None of the trials selected for this experiment were used in Experiment 1.

**Table 2:** Descriptive statistics of the images used in Experiment 2. Abbreviations: (1) target object's distance from threshold; (2) number of total objects in the image; (3) number of objects in the reference set (RS); (4) number of objects in the RS with the same size as the target object (target object included); (5) number of objects in the RS with a different size compared to the target object; (6) number of total colors in the image

	Min	Max	Mean ± *SD*
1. target *dft*	0.003	0.207	0.10 ± 0.06
2. #objs_IMG	5	9	6.69 ± 1.47
3. #objs_RS	3	6	3.94 ± 0.80
4. #sameS_IMG	2	5	2.44 ± 0.73
5. #diffS_RS	1	3	1.50 ± 0.67
6. #colors_IMG	3	5	4.09 ± 0.78

### 3.2 Method

**Fig. 4:**
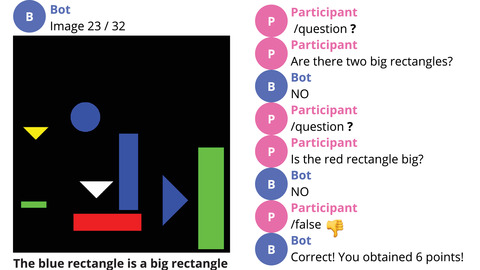
Example of a game trial in the Question (Q) condition, where the blue rectangle is *small* according to the bot's (MALeViC) interpretation. After asking two questions, the participant correctly assesses that the statement *The blue rectangle is a big rectangle* is *false* according to the bot. Best viewed in color.

We collect data by means of a game‐like script running on the cloud‐based instant messaging service Telegram.^9^ In the game, participants interact with a bot for 32 trials. Each trial consists of an image and a statement (see Fig. 4) extracted from the dataset as described above. The task of the participant is to judge whether the statement is true or false for that image according to the interpretation of the bot (see Section 3.1). Before starting the experiment, participants are told that the interpretation of the bot is consistent throughout the game. Moreover, they are told that some of the objects in the reference set count as big and some as small, and we provide examples that make explicit that there is always at least one big and one small object with the same shape as the target.^10^ For each correct response, participants receive 10 points; for each wrong response, zero points. The goal of the game is to obtain the highest possible score after having assessed all the 32 trials, which in turn determines participants' monetary compensation. After each assessment, participants receive automated feedback and are shown the number of points obtained for that trial along with the total score up to that moment. This feedback is crucial for the aim of the experiment, which aims at testing whether people can achieve communicative success with an interlocutor by adapting to its representations of *big/small*.

We consider two conditions: a Question (Q) condition where participants have the possibility to ask yes/no questions before assessing each trial, and a Control (C) condition where this possibility is not given. In the Q condition, asking questions incurs a penalty: for a correct assessment after one question, participants receive seven points; after a second question, six points, etc. The aim of the penalty is twofold: first, we mimic a real‐life scenario where asking a question has a cost both in terms of time and cognitive effort (Rothe et al., 2018); consistently, asking questions should not be used as a *default* strategy for all trials, but only when in doubt. Second, we want to ensure participants ask questions that are as useful as possible. Wrong assessments yield zero points in all cases.

In both conditions, participants are told that they will be interacting with an automatic bot that uses *big/small* consistently throughout the trials. In the Q condition, however, answers are sent by an experimenter through the messaging service. To make this *Wizard‐of‐Oz* scenario credible and facilitate the task of the experimenter, only polar questions are allowed—the bot/experimenter can only reply with *yes*, *no*, or *invalid*.^11^ The bot was implemented in Python using the python‐telegram‐bot library.^12^ It was run on a local machine and thus activated only during the data collection.

We collected valid data from 40 participants, 20 in the Q condition and 20 in the C condition.^13^ To exclude any effects due to the ordering of the trials, two orders were used. Each order was randomly selected and kept fixed for 10 participants in Q and 10 in C. To ensure that either order did not correlate with task difficulty, we considered the distance from the threshold of the target objects as an indicator of difficulty and checked that, on average, this value was constant through time. To do so, for each presentation order, we grouped adjacent trials in two or four groups containing 16 or eight trials each, respectively. We then tested whether there was any difference between the average *dft* of each group via a series of *t‐*test pairwise comparisons. No comparisons revealed reliable statistical differences (all p>.1).

In total, 1,280 datapoints (2 conditions × 2 orders × 10 participants × 32 trials) were collected. All participants were proficient in English and recruited among current or former university students and researchers. They read and signed an informed consent form before starting the experiment and were rewarded with a voucher worth a fixed amount plus a variable bonus based on their score. On average, participants received 4.75 € (∼9.5 € /h). No personal data besides their name, primary language, study program (if applicable), and contact information were collected. None of the 40 participants took part in Experiment 1.

### 3.3 Analysis and results

#### 3.3.1 People align via both passive and active interaction

As described above, participants were instructed to aim for the highest possible score. Due to the penalties applied in Q, however, the number of points obtained by the participants over the trials is not informative of speakers' alignment—measured in terms of their overall communicative success—in the two conditions. Instead, we can compare Q and C by considering the number of correct assessments participants made for each trial in each condition. In particular, for each trial in the two conditions, we count the number of total participants who provided the correct answer to it—with or without asking questions when given the possibility—and normalize it to range in [0,1]. We consider this normalized frequency as a proxy for the overall *alignment between participants and the bot*.

**Fig. 5:**
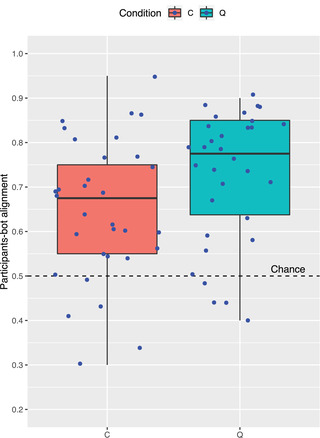
Distribution of participants–bot alignment in C and Q. Blue dots indicate the normalized frequency for each trial. Note that dots are slightly shifted from their actual value to avoid overlaps. Best viewed in color.

In Fig. 5, we report the distribution of alignment values in both C (M=0.65, SD=0.16) and Q (M=0.73, SD=0.15). As can be noted, the boxes in both conditions are above *chance* level. By means of a one‐sided Wilcoxon signed rank test, we verify that alignment in the Q condition (Mdn=0.78) is greater than 0.5: V=457, p<.001. Using a one‐sided one sample *t*‐test, we verify this is also the case for the C condition, where alignment is significantly higher than chance: t(31)=5.39, p<.001. This pattern is different from the one observed in Experiment 1, where the alignment between participants and the MALeViC interpretation was found not to be significantly greater than chance in *borderline* cases—though we acknowledge that this divergent pattern could be the result of a difference in power between the two experiments. These results are consistent with the hypothesis that participants, during the game, may *adapt* their semantic function (i.e., they would tune their *k*) to that by the target speaker based on the received feedback.

While alignment is significantly higher than chance in both conditions, it can be noted in Fig. 5 that the Q box is *above* the C one, which suggests an advantage of the active condition over the passive one. Indeed, the difference between C (*Mdn* = 0.68) and Q (*Mdn* = 0.78) turns out to be significant as per a one‐side non‐parametric Wilcoxon rank sum test, W=672.5, p=.015.^14^

**Fig. 6:**
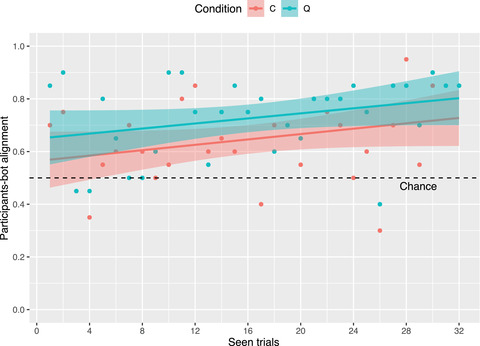
Alignment between participants and the bot increases over time in both C and Q as a result of semantic adaptation. Colored dots indicate normalized frequency in each condition. Best viewed in color.

As also reported in Fig. 6, in 20 trials out of 32 alignment is higher for Q than for C, while the opposite trend is observed only in six cases (a tie is observed for the remaining six cases). This is also supported by the observation that, on average, participants make a higher number of correct assessments in Q (M=23.30, SD=3.77) than in C (M=20.75, SD=1.83). Crucially, alignment between participants and the bot is observed to increase over time, which reveals that semantic adaptation is in place in both conditions. As indicated by the linear regression lines fitting the data in Fig. 6, alignment monotonically increases as the game progresses, which indeed reveals that adaptation is in place in both C and Q. Moreover, the striking similarity between the slopes of the two regression lines indicates that semantic adaptation has a comparable “speed” in the two conditions. That is, the extra advantage of asking questions over passive feedback is constant over time. It should be noted that all these observations hold when zooming into each of the two presentation orders in which data were presented (not reported).

Taken together, these results support our hypothesis that speakers adapt to an individual's interpretation of *big/small*, and this takes place both in the passive and active conditions. As hypothesized, semantic alignment between participants and the target speaker appears to be higher in Q than in C, which supports our hypothesis that participants do ask clarification questions in Q, and that this is beneficial for semantic adaptation. In the next section, we report the results of a statistical analysis aimed at more formally evaluating whether, and to what extent, experimental condition (Q vs. C), level of exposure to the target interpretations, and a number of perceptual factors play a significant role toward greater semantic alignment.

#### 3.3.2 Asking clarification questions improves semantic alignment

In this analysis, we aim to evaluate which factors lead to higher semantic alignment. We conjecture that, besides the possibility of asking questions, other factors such as the amount of exposure to a specific interpretation and the perceptual properties of the visual scene might play a role. We operationalize semantic alignment in terms of communicative success between a participant and the bot. In practice, we consider a trial as successful if the participant correctly assesses the statement; otherwise, we consider it as unsuccessful.

We experiment with the aggregated data (1,280 datapoints) and fit a mixed‐effect logit regression model (Baayen, Davidson, & Bates, 2008; Jaeger, 2008) to predict a binary output: success/unsuccess. We include 12 main variables: (1) experimental condition, that is, Q/C (factor); (2) target object's size category according to MALeViC, that is, *big/small* (factor); (3) target object's shape type (factor); (4) target object's color (factor); (5) total number of objects in the scene (integer); (6) number of objects in the reference set (integer); (7) number of objects in the reference set with same size category as the target one (integer); (8) number of objects in the reference set with different size category as the target one (integer); (9) number of unique colors present in the scene (integer); (10) order of trials' presentation (factor); (11) target object's distance from the threshold (numeric); (12) amount of exposure, that is, number of trials seen up to that moment (integer). As random effects, we included: participant *id* (factor); image *id* (factor). We tested a version of the model with no interactions between the main effects (*baseM*) and one including an interaction between (11) and (12) to evaluate the effect of perceptual difficulty in modulating the level of exposure to a specific interpretation (*intM*). For both models, we adopted a backward model simplification procedure: starting from the full‐factorial model including all the independent variables, we progressively simplified it by removing, at each step, the predictor with the largest *p*‐value, after checking its contribution with a likelihood ratio test  (Kuznetsova, Brockhoff, & Christensen , 2017). This simplification procedure ended when removing other variables would cause a reliable deterioration in model fit, that is, when the fit of the model *excluding* the target variable significantly differed (p<.05) from the fit of the model *including* it.

First of all, we verify that both the final *baseM* ($X^{2}\left(5\right)=37.48$, p<.0001) and *intM* ($X^{2}\left(6\right)=43.31$, p<.0001) perform significantly better than an intercept‐only model including no fixed effects. This is confirmed by the lower AIC values (Akaike, 1973) of both *baseM* (1471.9) and *intM* (1,468.1) compared to the null model (1,499.4). Second, we verify that adding the interaction term makes *intM* fit significantly better than *baseM* ($X^{2}\left(1\right)=5.83$, p<.016), which is also indicated by the lower AIC value. In what follows, we therefore focus on *intM* and discuss it in detail. In Table 3, we report the estimated coefficient (β) and standard error (*SE* β), *z*‐value (*z*), odds ratio (OR), confidence interval (CI), and *p*‐value (*p*) of each fixed effect included in the final model.^15^

**Table 3:** Logit including the interaction term (*intM*). For each fixed effect, we report estimated coefficient (β) and standard error (*SE* β), *z*‐value (*z*), odds ratio (OR), confidence interval (CI), and *p*‐value (*p*). *** stands for p<.001; ** p<.01; * p<.05; p<.1. Bottom: model's Akaike Information Criterion (AIC), Bayesian Information Criterion (BIC), deviance, and *R* ^2^

Predictor	β	*SE* β	*z*	OR	CI	*p*
(Intercept)	1.63	0.49	3.36	5.10	1.97–13.22	< .001${}^{***}$
Target object's size [small]	−0.93	0.23	−4.05	0.40	0.25–0.62	< .001${}^{***}$
Condition [Q]	0.43	0.16	2.77	1.54	1.14–2.09	.006${}^{**}$
Number of same‐size objects in RS	−0.41	0.15	−2.63	0.67	0.49–0.90	.009${}^{**}$
Number of seen trials (trials)	−0.01	0.02	−0.51	0.99	0.96–1.02	.609
Distance from threshold (dft)	1.28	2.76	0.46	3.58	0.02–801.06	.644
Trials * dft	0.34	0.14	2.40	1.41	1.06–1.87	.017*
AIC/BIC/deviance	1468.1/1514.5/1450.1					
Marginal *R* ^2^/conditional *R* ^2^	0.13/0.19					

First, the possibility of asking clarification questions, that is, the Q condition, is confirmed to be a highly reliable predictor of participants' semantic alignment (β=0.43, z=2.77, p=.006): the probability of achieving communicative success is higher for a trial in Q than in C. As signaled by the odds ratio, in particular, the odds of achieving communicative success (vs. being unsuccessful) for a trial in Q increase by a factor of 1.54 compared to a trial in C. This indicates that participants do ask questions when given the possibility (see Section 3.3.3) and that this provides extra information that leads to stronger feedback for adaptation compared to passive exposure. By asking questions, participants understand better where the *big/small* threshold is in their interlocutor's semantic representations (i.e., what *k* is used in their semantic function), which leads to higher semantic alignment.

A second reliable predictor is the size label of target objects (β=−0.93, z=−4.05, p<.001): in particular, objects that are annotated as *small* in MALeViC are more likely to lead to a wrong assessment compared to *big* ones. This shows that participants often consider as *big* objects that are *small* according to MALeViC. One possible explanation is that, when interpreting *big*, participants rely on a greater *k* value compared to the target one, that is, 0.29. As illustrated in Fig. 1, this would lead participants to consider a wider range of areas in the reference set's distribution as *big* compared to MALeViC. Alternatively, this could result from participants computing their probabilistic threshold based on the entire set of objects depicted in the image—not just on the objects with the same shape as the target one—which would lead to a slight bias toward using the *big* label.^16^

A third reliable predictor is the number of objects in the reference set bearing the same size category as the target object (β=−0.41, z=−2.63, p=.009): the higher the number of these objects, the more communication is likely to be unsuccessful. This effect suggests that participants are sensitive to the position of the target object in the ranking of object areas: the higher the number of objects in the reference set with a larger/smaller area, the less the target object is likely to be considered as *big/small*.

**Fig. 7:**
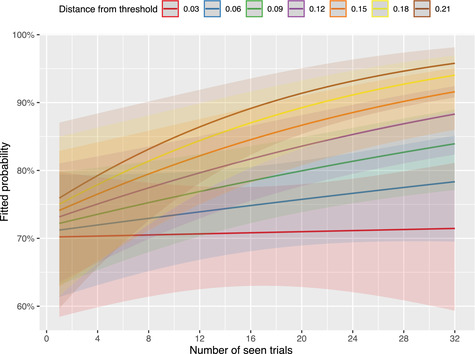
Plot showing the interaction between target object's distance from the threshold (*dft*) and number of seen trials. As can be seen, the role of experience with someone else's interpretation toward providing a correct assessment is stronger when *dft* is high. Best viewed in color.

Finally, we found a statistically reliable interaction between the number of seen trials (which is a proxy for the amount of exposure to a specific interpretation) and target object's distance from threshold (β=0.34, z=2.40, p=.017): the number of seen trials is more powerful in predicting communicative success when the distance from the threshold is higher (see Fig. 7). This indicates that experience of an interlocutor's interpretation—both in a passive and active interaction—plays a big role toward adapting to it when the object's area is relatively far from the threshold. At the same time, this role is weaker when distance from threshold is extremely low; in these cases, speakers might encounter extra difficulties due to an inexact estimation of objects' areas, which suggests that adaptation cannot fully overcome perceptual difficulties.

Seven variables did not make it to the final model. In particular, neither the shape type nor the color of the target object was found to be reliable predictors, which indicates that participants genuinely focused on the area rather than other perceptual features of the geometric objects. Consistently, the number of unique colors present in the image was also excluded. As for the exclusion of the number of objects in both the scene and the reference set, as well as the number of objects in the reference set with a different size than the target object, this reveals that participants' assessments were not affected by the number of objects under consideration. Finally, the order of presentation of the trials did not play any statistically reliable role, which confirms that no significant differences with respect to the distribution of perceptually challenging trials in the two orders were present.

Overall, the results of the statistical analysis indicate that exposure to the target interpretation and the possibility of asking questions make people adapt toward an interlocutor's interpretation of *big/small*, which leads to improved semantic alignment. At the same time, adaptation does not fully overcome perceptual difficulties which are due, for example, to the presence of many objects with the same shape and size or to an inexact estimation of objects' areas. Crucially, alignment is found to be higher when participants are given the possibility to ask clarification questions to their interlocutor.

#### 3.3.3 Not all questions are equally informative, and people learn to ask better ones over time

Our results so far suggest that speakers ask clarification questions, and that this has a positive effect toward achieving communicative success. In total, participants in Q ask questions in 213 datapoints out of 640. Out of these question *sequences*, 194 include one ⟨question,answer⟩ pair, and 19 two or more. This number reveals that participants, on average, ask questions in around 33% cases. Therefore, participants do ask questions if given the possibility but not for all the trials.^17^ This is consistent with the fact that asking questions is not costless, and people do not overuse this communicative tool when not strictly necessary.

We then analyze question *success*: if a participant makes a correct assessment after asking one or more questions and receiving the corresponding answer(s), then we consider the entire question sequence as successful; otherwise, we count it as unsuccessful. Out of 213 question sequences, 187 are successful (88% of total), while 26 are not (12%). Thus, while the vast majority of question sequences lead to success, this is not the case for all of them. As noted by Rothe et al. (2018), this is likely due to the level of *informativeness* provided by a ⟨question,answer⟩ pair, namely, the amount of relevant information it elicits. Consider the example in Fig. 4. Here, participants are asked to assess whether the blue rectangle is a *big* rectangle. In this case, asking the question *Are there two big rectangles?* will be highly informative if the answer is *yes* (since the blue rectangle has a larger area than the red one, we will know for sure that the statement is true)^18^; in contrast, if the answer is *no*, there might still be either just one or three big rectangles, and the question–answer pair will not be fully informative (i.e., a second question will be needed). In a similar setting, Rothe et al. (2018) found that participants rarely asked highly informative questions, which indicates that maximizing the utility of questions is a challenging task.

We manually evaluate the informativeness of the questions asked by the participants in our task against the corresponding visual scene. We opt for a simple binary categorization and annotate a question sequence as *resolutive* if no uncertainty is left after receiving the last answer; *non‐resolutive* if some degree of uncertainty is still present.^19^ This annotation was performed by one of the authors using the metadata provided in MALeViC.

**Fig. 8:**
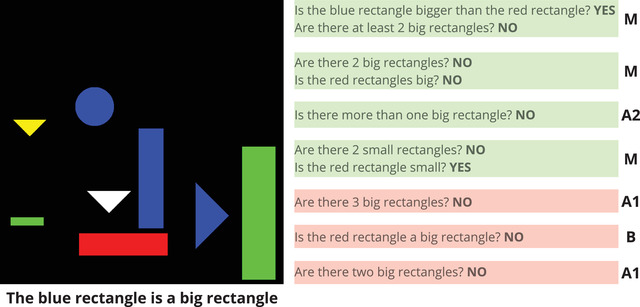
All resolutive (green) and non‐resolutive (red) question sequences produced for one trial in Q. Note that the statement is *false*. The letters on the right stand for the annotation on sequence question type (see Section 4 and Table 5). Best viewed in color.

Fig. 8 reports all the question sequences produced by participants in Q for one specific trial (the same of Fig. 4). Resolutive question sequences are highlighted in green, non‐resolutive ones in red. As can be seen, three sequences—the first, second, and fourth—include two ⟨question,answer⟩ pairs: the first sequence is resolutive based on the second pair only, while both the second and the fourth are resolutive due to the combined information brought by the two ⟨question,answer⟩ pairs they contain; that is, none of the pairs in these sequences are resolutive on their own. The third example, in contrast, includes only one pair which turns out to be resolutive on its own. As for the last three question sequences, they include one ⟨question,answer⟩ pair and are all annotated as non‐resolutive. In fact, none of them completely exclude the possibility that the big rectangles may be still either two (fifth and sixth example) or three (seventh example) and not just one, which is the correct scenario in this case. As a consequence, they still make it possible that the statement is *true* according to the target speaker, which is wrong in this case.

The results of this annotation are reported in Table 4. First, resolutive question sequences turn out to be almost twice as frequent as non‐resolutive ones: 138 versus 75, which indicates that participants are often able to ask questions that, together with their answers, potentially lead to communicative success. However, non‐resolutive ones still account for 35% of total question sequences, which confirms that, in more than one third of cases, people ask questions that do not guarantee a correct assessment. Second, as expected, resolutive sequences have a virtually perfect success rate (0.99; see Table 4). In contrast, non‐resolutive ones do not help to better discover the interlocutor's semantic function compared to receiving passive feedback: their success rate is equal to (0.67), which is basically on par with the success rate in C (0.65) and in the cases of Q where no questions are asked (0.65). This indicates that resolutive questions that remove all uncertainty regarding the context play a quantifiable, significant role toward improving communicative success over the passive condition, while non‐resolutive ones do not—or do it only marginally. At the same time, it is worth mentioning that in no case is asking questions harmful to semantic adaptation. This suggests that non‐resolutive questions have some utility—at the very least toward learning what counts as a good question—whose operationalization deserves further investigation.

**Table 4:** Number of cases in C and Q by type of question (where applicable) and corresponding success rate normalized in range [0,1]. The bottom row reports total cases and success rate per condition

	C			Q		
	Cases	Success cases	Success rate	Cases	Success cases	Success rate
No questions	640	415	0.65	427	279	0.65
*Resolutive* questions	–	–	–	138	137	0.99
*Non‐resolutive* questions	–	–	–	75	50	0.67
Total	640	415	0.65	640	466	0.73

**Fig. 9:**
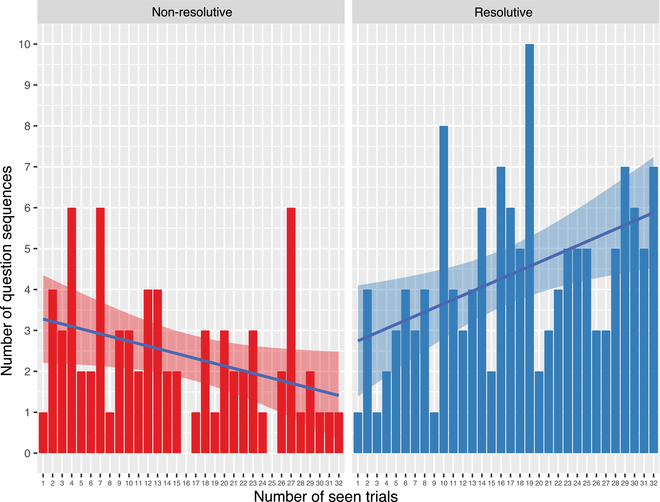
Total number of non‐resolutive (left) and resolutive (right) question sequences. The former decrease over time (number of seen trials), the latter increase. Best viewed in color.

We conjecture that participants might learn to ask more resolutive questions as the interaction progresses. Our hypothesis is that experience with the task and the utility of various questions should make participants increasingly more efficient. That is, they would move from questions that may have little or perhaps indirect utility to questions that would ensure communicative success by directly confirming or disconfirming a hypothesis. To test this, we compute Spearman's correlation (ρ) between the total number of resolutive question sequences asked by participants for each trial and the number of trials seen until that point. We find a substantial positive correlation (ρ=0.50, p=.004). To check whether this effect is due to a generalized increase in the number of *any* type of question, we perform the same analysis between trials seen and the total number of question sequences per trial. In this case, no significant correlation is found (ρ=0.18, p=.332), which reveals that only more resolutive questions, but not questions in general, are asked as the interaction progresses. This could imply that, while resolutive question sequences increase, non‐resolutive ones decrease over time. To test this possibility, we compute Spearman's correlation between seen trials and the proportion of resolutive sequences (resolutive/total) per trial. We find a rather strong positive correlation (ρ=0.54, p=.002), which confirms that, as the interaction progresses, resolutive question sequences increase while non‐resolutive ones decrease (see Fig. 9).

In the next section, we perform a linguistic analysis aimed at exploring what are the most frequent questions asked by people and whether certain types of questions are more informative than others.

## 4 Linguistic analysis of questions

We analyze the type of questions participants ask during the interaction. We examine all the questions asked and identify four types of questions: (**A**) questions with quantifiers, used to ask about the number of objects belonging to a given size class (*Is/Are there exactly N/only N/at least N/more than N/any big/small* shape *?*); (**B**) questions asking about the size label of an object in the reference set (*Is the* color shape *big/small?*); (**C**) questions with a comparative form used to ask about the size of two objects relative to each other (*Is the* color shape_1_ *bigger/smaller than the* color shape_2_ *?*); (**I**) questions that are either not polar or directly ask about the size label of the target object and are therefore invalid.

We annotate each question sequence with the type of question(s) present in the sequence. If a sequence includes one single valid question or more than one valid question of the same type (types **A**, **B**, or **C**), then the whole sequence is annotated with that question type. Question sequences that include more than one type of valid question are annotated as mixed (**M**). No question sequences include only invalid questions. Some examples of annotated sequences are available in Fig. 8.

For each type of question sequence, in Table 5 we report a templated description along with its frequency, the count and proportion of its resolutive cases, and the number of participants who use it at least once during the game. For **A** sequences, the statistics are broken down per type of quantifier.

**Table 5:** Manual annotation of question sequence types. We report frequency (f), number (%) of *resolutive* cases (res), and number of participants (p) using it at least once

Type of question		*f*	res (%)	p
**A**	Questions with quantifiers	171	134 (78%)	19
	**A1** Is/are there *exactly N* big/small shape?	89	66 (74%)	8
	**A2** Is/are there *more than N* big/small shape?	32	32 (100%)	4
	**A3** Is/are there *at least N (N>1)* big/small shape?	23	23 (100%)	1
	**A4** Is/are there *only N* big/small shape?	14	13 (93%)	2
	**A5** Is/are there *any* big/small shape?	13	0 (0%)	9
**B**	Is *the* color shape (a) big/small (shape)?	32	0 (0%)	12
**C**	Is *the* color shape_1_ bigger/smaller than *the* color shape_2_ ?	5	0 (0%)	3
**M**	Mixed: more than one valid question type	5	4 (80%)	4

Several observations can be made. First, most questions asked are quantified questions (type **A**). This is the case when considering both the overall frequency of **A** questions (171 out of 213 total sequences) and the number of participants who use them (19 out of 20). In particular, **A1** is the most‐occurring question type (89 occurrences) with an almost triple frequency compared to **A2** and **B**, the second most frequent types (32 occurrences each). This indicates that asking for confirmation of the number of shape types with a certain size (e.g., *Are there two big rectangles?* ^20^) is both a common and highly iterated strategy: participants who use it, on average, resort to it more than 11 times during the game.

Second, only question sequences with quantifiers (types **A** and **M**) are resolutive. For example, **A1** is resolutive in around three out of four (74%) cases, which makes it a fairly reliable strategy to achieve communicative success. **A2**, **A3**, and **A4** can be considered as a refined version of **A1**: by means of a modifier of the numeral (*more than N*, *at least N*, and *only N*), these question types have an even greater chance to be resolutive. However, only a few participants were able to ask these questions, which confirms this ability is not trivial since it requires a careful evaluation of the potential information gain brought by each question in the question space (see Rothe et al., 2018). Accordingly, participants stick to one question type if it is highly rewarding: see, for example, the somehow extreme case of **A3**, which is used 23 times by the same participant over the game.

Third, some question types are never resolutive: this is indeed the case for **A5**, **B**, and **C**. Some of these types are asked by a fair amount of participants, in particular, **A5** and **B** (with 9 and 12 participants, respectively), and appear to be exploratory. In fact, **A5** provides information that should already be clear to participants, namely, that there is at least one *small* and one *big* object in the reference set. We observe that most of these question types are asked in the first half of the trials: 75% of **A5** and 63% of **B**, which supports the hypothesis that participants use them as an exploratory strategy.

Besides being informative of how (often) participants use certain question types, this analysis more broadly suggests that individual differences in both the number of questions asked and their success over time are to be expected. Future work might further explore whether different patterns lead to different individual behaviors, and how this interacts with communicative success.

## 5 General discussion

Our study brings novel empirical evidence that speakers may disagree on whether a certain gradable adjective applies to an object in a given context due to the (slightly) different threshold functions employed and that, during linguistic interaction, speakers align their interpretations to those of their interlocutor to resolve such disagreement and achieve communicative success. This process of semantic *adaptation* takes place in a passive interaction setting but is constantly and significantly stronger when people can actively seek information by asking clarification questions. We observe that asking questions is a non‐default strategy, which is used by speakers only in the presence of dubious cases to obtain direct evidence on the interlocutor's interpretation. We also report that not all question–answer pairs are equally informative, and people become better questioners as the interaction progresses. Overall, our findings indicate that asking clarification questions is a powerful linguistic device that, despite its costs, plays a direct, quantifiable role toward discovering an interlocutor's semantic representations and achieving communicative success.

In Experiment 2, we focused on cases that are deemed *borderline* by one specific semantic function. This was operationalized in terms of the distance from the threshold computed by the semantic function by Schmidt et al. (2009): the lower the distance from the threshold, that is, the big/small boundary, the more likely is the object to be deemed borderline by the target threshold function. This approach may arguably have some limitations, which we discuss below.

First, it assumes that objects that are close to the threshold are still either *small* or *big*, with no buffer zones where neither of the two, but perhaps a third size category applies: for example, *regular* or *medium*. As described in Section 2, this is a consequence of the binarization proposed in the MALeViC data (Pezzelle & Fernández, 2019), which simplistically refers to non‐*big* objects as *small*. While other approaches could define *big* and *small* based on two (possibly different) semantic functions—which, in turn, would lead to other size labels being used—this would not eliminate borderline cases: regardless of the function being used, some objects will still be closer to the threshold than others are. As a consequence, speakers may still disagree on whether a certain adjective applies to a given context and need to adapt to an interlocutor's interpretation to achieve communicative success.

Second, with the threshold being computed based on the areas (in pixels) of the smallest, biggest, and target object in the image (see Section 2.1), this operationalization is naturally dependent on the accuracy with which a visual input is processed. In other words, even if participants were aware of the formula subtending the definition of the big/small threshold, this does not guarantee they will not make any errors due to an inexact estimation of the areas of the objects in the reference set (this is also discussed in Qing & Franke, 2014b). We believe this is also one of the reasons why communicative success, which significantly improves as a result of semantic alignment, does not become perfect. While this issue might be present to a lesser extent in other semantic functions, estimating object areas appears to be crucial for any probabilistic interpretation that is dependent on the visual context. As such, we expect disagreement not to completely disappear by virtue of the availability of a context (Van Deemter, 2012).

Third, the present approach defines borderline cases based on a single semantic function (and *k*), namely the best‐predictive one by Schmidt et al. (2009). While this approach is to some extent simplistic, it has the advantage of relying on a unique, fixed criterion that is not affected, for example, by the number of semantic functions considered and their (potentially different) level of plausibility (see also Solt & Gotzner, 2012). We leave to future work a more formal investigation of other semantic functions (including other values of *k*) in the context of semantic adaptation.

As for our experimental choices, in Experiment 2 we penalized participants who asked questions by subtracting some points from their score. As explained in Section 3, this was aimed to mimic real‐life scenarios where asking questions has a cost in terms of time and cognitive effort. Overall, this experimental choice seemed appropriate: participants in Q asked questions in around one third of trials, while making a guess in the remaining two thirds. However, we are aware that a different—lower or higher—penalty might lead to different patterns, with people asking more or less questions. We plan to investigate the impact of this choice in future work.

Several interesting questions are left open. First, one natural further question—not explored here—is whether adaptation can occur simultaneously with respect to several speakers entertaining various, potentially different interpretations. In our setting, this could be tested, for example, by having participants interact with several bots, each using a different semantic function (Schmidt et al., 2009; Solt & Gotzner, 2012). This would allow us to explore whether, and to which extent, adaptation takes place independently from the various semantic functions being used, and how this process interacts with the lexical properties of gradable adjectives that set constraints on their general applicability (see Kennedy & McNally, 2005).

Second, while our results show that asking questions improves semantic alignment and thus communicative success, we do not observe increased adaptation over time. Indeed, the advantage of the active interaction setting over the passive one is found to be constant throughout the trials, as is the number of questions asked per trial. Furthermore, we observe the same trend in the control and question conditions regarding the ratio of correct answers in trials where no questions are asked. This could be a consequence of the relatively limited number of trials used in our experiment (32). With a higher number of trials, the gap between the two conditions might increase and the number of clarification questions might decrease over time—a possibility we plan to investigate in future work. Our current results, however, suggest that interaction may not necessarily lead to increased long‐term adaptation. Indeed, the key advantage of conversational interaction may be to boost ad hoc coordination without committing to long‐term semantic updates.

Third, while our study shows that adaptation to a specific speaker takes place in the context of a specific, close‐ended interaction, it does not investigate how adaptation relates to learning: Does asking questions play also a role in the learning of our own semantic interpretations of words? To test this hypothesis, an experimental setting similar to ours could be used where participants are exposed to and interact with *novel* words whose meaning is grounded in an image (Amato & MacDonald, 2010; Fedzechkina, Newport, & Jaeger, 2016).

In future work, our method and findings can inform research aimed at devising probabilistic models that predict the optimal use of gradable adjectives based on a speaker's *pragmatic reasoning* over a context (Qing & Franke, 2014a, 2014b). In particular, the role of informative questions toward updating a speaker's beliefs regarding the interpretation of gradable adjectives could be used as one of the predictors, perhaps in conjunction with the time spent to come up with a question over the experiment or a pragmatic‐based estimation of the utility of a question, similar to Rothe et al. (2018). Moreover, our study can inform computational work on question generation in the domain of natural language processing (see, e.g., Wang and Lake, 2021). Asking (informative) questions could also be of crucial importance, for example, to multimodal AI models asked to provide a correct answer to a question regarding the content of an image (Antol et al., 2015; Johnson et al., 2017) or the abstract relation tying various scenes depicting similar objects (Parfenova, Elliott, Fernández, & Pezzelle, 2021). This possibility could reduce the uncertainty of a model—when the input question is vague, ambiguous, or can be misinterpreted—and drive its decisions toward the correct output.

## 6 Conclusion

We investigated whether semantic adaptation is in place for vague and context‐dependent gradable adjectives *big* and *small* and found that, in line with previous work experimenting with similar expressions (S. Heim et al., 2020; Schuster & Degen, 2019; Yildirim et al., 2016), people align to an interlocutor's interpretation—to achieve communicative success—by being exposed to it through linguistic interaction. From a broad perspective, this confirms that communication is one of the most important functions of language and that communicative context bridges the gap between the processing of language and speakers' intent (Hasson, Egidi, Marelli, & Willems, 2018).

In addition, we showed that asking clarification questions in the presence of dubious cases further improves alignment, while not all the question–answer pairs are equally informative. On the one hand, this confirms that information seeking plays a positive role at the semantic level since it helps understand the function governing an interlocutor's interpretation of *big* and *small*. On the other hand, the finding that speakers become better questioners as the interaction progresses indicates that asking maximally informative questions is not a trivial ability (Rothe et al., 2018), which improves with experience of the task and the interlocutor.

These results provide novel evidence that active information seeking plays a crucial role toward discovering latent structures of language besides serving as a core cognitive and linguistic tool to gain information about the world (Hawkins et al., 2015; Ruggeri et al., 2016). This paves the way for future work exploring the extent to which these findings apply to other levels of language analysis; for example, whether asking informative questions strengthens alignment at the phonetic level, where adaptation via passive exposure has been already observed. Building on our findings, such studies may shed new light on the way in which language can be shaped by communication, of which asking questions can be seen as the quintessence.

## Notes

1 This does not apply to non‐gradable adjectives: for example, if Galadriel is *immortal*, it follows that Galadriel is an *immortal* elf, an *immortal* Tolkien's character, etc. (Partee, 1995).

2 Materials, data, and analysis code to reproduce the results described in the paper are available here: https://surfdrive.surf.nl/files/index.php/s/IUi4X4QGkUiqm9Z

3 Here, we make the simplified assumption that all speakers' interpretations can be described in terms of the function by Schmidt et al. (2009), T=Max−k(Max−Min), though with possibly different values of *k*. This is similar to assuming that all speakers' interpretations of, for example, *few*, are based on proportions, while the actual proportion can vary between individuals (S. Heim et al., 2020; Schuster & Degen, 2019; Yildirim et al., 2016).

4 As reported by Pezzelle and Fernández (2019), *k* values are sampled from a distribution with μ=0.29 and σ=0.066. This is to avoid that *k* values exceed 0.5, that is, the proportion in the middle of the range of sizes. Indeed, using σ=0.066 theoretically ensures that 99.7% of the sampled *k* values will be in the range 0.09–0.49, that is, ±3 *SD* from 0.29.

5 See Pezzelle and Fernández (2019) for further details.

6
https://www.google.com/forms/about/

7 We opted for a non‐parametric test after observing a non‐normal distribution for one of the two independent samples. This was tested via a Shapiro–Wilk normality test, which was significant, *W* = 0.813, p=.021.

8 We first verified, via a Shapiro–Wilk normality test, that the data in the *borderline* sample do not significantly deviate from a normal‐like distribution, W=0.905, p=.251. As for *clear‐cut* trials, the same test revealed an opposite pattern, W=0.813, p=.021.

9
https://telegram.org/

10 The examples explicitly refer to the relevant reference set, for example: *The red rectangle is big given the other rectangles*; *Among the triangles, two are small and one is big*, etc.

11 The answers provided by the experimenter are strictly based on the MALeViC annotation. Questions involving numbers, for example, *Are there two big rectangles?*, are consistently treated as having an *exact* reading, that is, *exactly two*, unless they are preceded by a modifier (at least *N*, more than *N*, etc.). The answer *invalid* is used when the question is about the size label of the target object or is not polar.

12
https://github.com/python‐telegram‐bot/

13 Data from other three participants were discarded due to (1) a technical issue; (2) the participant not following the task instructions; (3) the participant not completing the game.

14 This test was performed after verifying that Q significantly deviates from a normal‐like distribution as per a Shapiro–Wilk normality test, W=0.888, p=.003. In contrast, this is not the case for C, W=0.975, p=.644.

15 Variance; *SD* of the random effects: participant *id* (0.07; 0.27), image *id* (0.17; 0.41).

16 For six out of 32 trials, computing the threshold at the image level would lead to using an opposite adjective for the target object as compared to the MALeViC one. In particular, the label would switch from *small* to *big* in four out of six of such cases, which is consistent with the observed trend.

17 Interestingly—as revealed by a number of correlation analyses—participants are not significantly driven by an object's *dft* when asking a question. This suggests that the reasons for asking a question about a given object may lie in other perceptual or social aspects related to the trial or the task. Further work is needed to explore this issue.

18 Recall that task instructions make clear to participants that, in the reference set, there is at least one *big* and one *small* object.

19 The degree of utility of *non‐resolutive* questions could be further measured using complementary approaches based, for example, on Bayesian methods (Rothe et al., 2018).

20 Since the experimenter consistently answered all questions including numbers as having an *exact* reading (*exactly N*), we annotate them accordingly.
